# Impact of Genome‐Wide and Regional Inbreeding on Semen Production Traits in Beef and Dairy Bulls

**DOI:** 10.1111/asj.70138

**Published:** 2025-12-03

**Authors:** Rintaro Nagai, Masashi Kinukawa, Toshio Watanabe, Atsushi Ogino, Kazuhito Kurogi, Kazunori Adachi, Yoshinobu Uemoto

**Affiliations:** ^1^ Graduate School of Agricultural Science Tohoku University Sendai Miyagi Japan; ^2^ Maebashi Institute of Animal Science Livestock Improvement Association of Japan Inc. Maebashi Gunma Japan; ^3^ Cattle Breeding Department Livestock Improvement Association of Japan Inc. Koto Tokyo Japan

**Keywords:** age of inbreeding, Holstein bulls, inbreeding depression, Japanese Black bulls, run of homozygosity

## Abstract

To evaluate inbreeding depression, which is a reduction in the phenotypic mean of a population due to mating between close relatives, for semen production traits in cattle, it is necessary to understand inbreeding depression both regionally and across the genome. This study aimed to investigate genome‐wide and regional inbreeding depression in semen production traits in Japanese Black bulls (JB) and Holstein bulls (HOL). The present study used 615 JB and 873 HOL with a 50K single‐nucleotide polymorphism (SNP) BeadChip and five semen production traits. First, we estimated the regression coefficients of the semen production traits on pedigree‐based and genomic inbreeding coefficients. Our results indicated significant negative effects of inbreeding on four semen production traits in JB, whereas only one trait was significantly affected in HOL. Second, we performed genome‐wide association studies to identify run of homozygosity (ROH) regions that had an unfavorable effect on the traits. Our results showed no significant SNPs in JB; however, SNPs on chromosomes 7 and 17 were significantly associated with sperm quality in HOL. This study revealed that semen production traits are influenced by genome‐wide and regional inbreeding depression in JB and HOL, although the effects vary depending on the target population.

## 1 Introduction

Inbreeding depression is defined as a reduction in the population mean for fitness‐related traits that occurs as a result of mating between close relatives. This phenomenon occurs from harmful recessive mutations in the homozygous state (Caballero 2020; Charlesworth and Willis 2009). Inbreeding depression has traditionally been investigated by regressing individual performance on pedigree‐based inbreeding coefficients (Caballero 2020; Charlesworth and Willis 2009; Curik et al. 2001). Several studies have investigated inbreeding depression in livestock. Inbreeding has an unfavorable effect on many traits, including reproductive and performance traits, with a 1% increase in pedigree‐based inbreeding coefficient associated with a median decrease in phenotypic value of approximately 0.13% of a trait's mean (Doekes et al. 2021; Leroy 2014). In addition, inbreeding is unavoidable in closed cattle breeding populations, such as Japanese Black cattle, which are a major beef breed in Japan and known for their high marbling (Gotoh et al. 2014). High selection intensity for marbling leads to increased inbreeding and a decline in effective population size (*N*_e_) in Japanese Black cattle (Nomura et al. 2001). Understanding how inbreeding affects fitness‐related traits is crucial for managing genetic selection in cattle populations.

Inbreeding coefficient is a measure of inbreeding level and is traditionally calculated using pedigree information. Nowadays, because of advances in genotyping technologies, high‐ or middle‐density single‐nucleotide polymorphism (SNP) arrays are available at low cost for cattle. Consequently, pedigree‐based inbreeding coefficients can be replaced with genomic inbreeding coefficients. Genomic inbreeding coefficients are unaffected by pedigree completeness and pedigree errors (Pryce et al. 2014). In addition, considering the Mendelian sampling effect, genomic inbreeding coefficients are expected to be more accurate than pedigree‐based inbreeding coefficients (Howard et al. 2017). Genomic inbreeding coefficients are generally derived from the diagonal elements of the genomic relationship matrix (GRM) or from the proportion of the genome covered by homozygous segments. Continuous homozygous segments of the DNA sequence are generally explained by run of homozygosity (ROH). ROHs are defined as contiguous homozygous stretches in an individual genome resulting from the transmission of identical haplotypes from parents to offspring (Broman and Weber 1999; McQuillan et al. 2008). These stretches can occur as a result of inbreeding. The evaluation of inbreeding using ROH information has the advantages of both proving genome‐wide inbreeding and identifying genomic regions underlying inbreeding depression (Ferenčaković et al. 2017; Ghoreishifar et al. 2023; Pryce et al. 2014; Scott et al. 2025). In addition, ROH length can be used to estimate the age of inbreeding (Ceballos et al. 2018; Keller et al. 2011).

Inbreeding has an unfavorable effect on many quantitative traits, including bull fertility in cattle (Doekes et al. 2021; Leroy 2014). The bull conception rate (BCR) is the percentage of achieving pregnancy and is the most important indicator of bull fertility. On the other hand, bull fertility has generally been evaluated using semen production traits such as semen quality and quantity. This is because strong genetic correlations have been reported between semen quality and BCR in Japanese Black bulls (JB) (Uemoto et al. 2024), and between semen quality and nonreturn rate in Holstein bulls (HOL) (Gebreyesus et al. 2021). In addition, the number of semen straws obtained from a bull directly affects the profitability of artificial insemination (AI) centers. Therefore, genetic improvement of semen quality and quantity in bulls is of interest, and understanding how inbreeding affects semen production traits is essential to effectively manage genetic selection in cattle populations.

To evaluate inbreeding depression for semen production traits in cattle, it is necessary to clarify the relationships of both genome‐wide inbreeding levels and homozygous regions within the genome with semen production traits. However, only a limited number of studies have reported the genomic inbreeding depression for semen production traits in both regional and across the genome in dairy cattle populations (Ferenčaković et al. 2017; Ghoreishifar et al. 2023). Therefore, the objective of this study was to investigate genome‐wide and regional inbreeding depression in semen production traits in JB as beef cattle and HOL as dairy cattle. First, we estimated the effect of genome‐wide inbreeding on semen production traits. Second, we performed ROH‐based genome‐wide association studies (GWAS) on semen production traits, accounting for different lengths of ROHs, to identify ROH regions underlying inbreeding depression.

## 2 Materials and Methods

### 2.1 Population and Data Collection

The population, phenotypes of semen production traits, pedigree, and genotypes of JB and HOL were resourced from Livestock Improvements Association of Japan Inc. (LIAJ) and the Holstein Cattle Association of Japan (HCAJ), which were identical to those described by Atagi et al. (2017) and Nagai et al. (2022a, 2022b). These datasets were used in this study. In brief, semen production records were obtained from four AI centers of LIAJ, and the records were collected from 1990 to 2020. Five semen production traits were evaluated: semen volume (VOL, mL), sperm number (NUM, $\times 10^{8}$), sperm concentration (CON, $\times 10^{8}/\mathrm{mL}$), sperm motility (MOT, %), and MOT after freeze–thawing (aMOT, %). Bulls were selected according to the following criteria: JB born between 1991 and 2012 and HOL born between 1989 and 2014, bulls with at least 20 semen production records, and bulls with both SNP genotypes and pedigree records. Semen production records were selected according to phenotypic values (within mean ± 4SD), bull's age at collection (12–96 months), and at least 20 records in each subclass of the contemporary group (defined by “year‐season‐AI center”). The number of selected semen production records was 65,463 for 615 JB and 50,734 for 873 HOL, which were the same as those described by Nagai et al. (2022a). Descriptive statistics for these traits are shown in Table S1. In addition, pedigree records of 3713 JB and 3839 HOL traced back to four generations were obtained from LIAJ and HCAJ, respectively.

Genotypic records for JB and HOL, which were the same records in Nagai et al. (2022a), were obtained from the LIAJ and HCAJ databases, respectively. Records were obtained using an Illumina BovineSNP50 BeadChip (v1 and v2; Illumina, San Diego, CA, USA). All SNP positions were updated according to the SNPchiMp v.3 database (Nicolazzi et al. 2015), and the ARS‐UCD1.2 reference sequence assembly was downloaded from Ensembl (release97, http://ftp.ensembl.org/pub/release‐97/variation/vcf/bos_taurus/). Missing genotypes were imputed using the FIMPUTE v3 software (Sargolzaei et al. 2014). After imputing missing genotypes, SNP quality control was assessed using PLINK 1.9 software (Purcell et al. 2007). The exclusion criteria for SNPs were the Hardy–Weinberg equilibrium test with a *p* value < 0.001 and the use of autosomes. A total of 48,642 SNPs in 615 JB and 45,950 SNPs in 873 HOL across 29 *Bos taurus* autosomes (BTAs) were available in the initial dataset.

### 2.2 Pedigree‐Based and Genomic Inbreeding Coefficients

In this study, one pedigree‐based inbreeding coefficient and five genomic inbreeding coefficients were calculated to estimate the genome‐wide inbreeding depression. Pedigree information, which was traced back to four generations, was used to calculate the pedigree‐based inbreeding coefficient ($F_{\mathrm{PED}}$) using the algorithm described by Meuwissen and Luo (1992). For genomic inbreeding coefficients, we defined five different genomic inbreeding coefficients derived from the GRM ($F_{\mathrm{GRM}}$) and homozygous segments ($F_{\mathrm{ROH}2}$, $F_{\mathrm{ROH}2-8}$, $F_{\mathrm{ROH}8}$, and $F_{\mathrm{HBD}}$).

$F_{\mathrm{GRM}}$ was derived from VanRaden's GRM (VanRaden 2008), which was calculated as follows:

$$G=\frac{{\mathrm{ZZ}}^{\prime }}{2\sum_{j=1}^{m}p_{j}\left(1-p_{j}\right)},$$

where *m* is the number of SNPs, $p_{j}$ is the frequency of the second allele of the *j*th SNP and is fixed at 0.5 in all loci, and the elements of **Z** are calculated as $z_{\mathrm{ij}}=x_{\mathrm{ij}}-2p_{j}$, where $x_{\mathrm{ij}}$ is the number of second alleles of the *i*th individual at the *j*th SNP. The diagonal elements of G minus 1 are defined as $F_{\mathrm{GRM}}$. $F_{\mathrm{GRM}}$ has been employed to monitor genetic diversity in dairy cattle at the Animal Genomics and Improvement Laboratory of the USDA (Guinan et al. 2023). In the calculation of $F_{\mathrm{GRM}}$, SNPs were assessed using the exclusion criterion of minor allele frequency (MAF) < 0.01, and 36,983 SNPs for JB and 38,364 SNPs for HOL were identified.

$F_{\mathrm{ROH}2}$, $F_{\mathrm{ROH}2-8}$, and $F_{\mathrm{ROH}8}$ are based on ROH segments. In this study, ROH was estimated using PLINK 1.9 software (Purcell et al. 2007), which uses a sliding window approach to define ROH as a stretch that includes a minimum specified number of homozygous SNPs within a specified distance. The following parameters and thresholds were applied to detect an ROH: (i) the number of heterozygous SNPs allowed in the ROH was 1; (ii) the maximum allowed distance between consecutive SNPs was 1 Mb; (iii) the minimum density of SNP in a sliding window was 1 SNP every 100 kb; (iv) the minimum length of an ROH was set to 2 Mb; and (v) the sliding window size and the minimum number of consecutive homozygous SNP included in the ROH were *L*, which was calculated in each breed as follows:

$$L=\frac{\ln\frac{\alpha }{n_{s}n_{i}}}{\ln\left(1-\bar{\mathrm{het}}\right)},$$

where *α* is the false positive probability set to 0.05, $n_{s}$ is the number of SNPs per individual, $n_{i}$ is the number of genotyped individuals, and $\bar{\mathrm{het}}$ is the means of heterozygosity across all SNPs (Lencz et al. 2007; Purfield et al. 2012). Genomic inbreeding coefficients based on ROH were defined as the total length of the ROH divided by the overall length of the autosomal genome (McQuillan et al. 2008). The overall length of the autosomal genome was defined as 2,489,385 kb based on the ARS‐UCD 1.2 reference sequence assembly (https://www.ncbi.nlm.nih.gov/datasets/genome/GCF_002263795.1/).

To evaluate the effect of ROH length on inbreeding depression, we grouped the ROH classes based on the age of inbreeding. Specifically, we grouped inbreeding based on ROH shorter than or longer than 8 Mb to create an “ancient” and a “recent” ROH (abbreviated to ROH2–8 and ROH8, respectively), from the entire length of ROH (ROH2) (Lozada‐Soto et al. 2021). The genomic inbreeding coefficients relevant to the different length classes were defined as $F_{\mathrm{ROH}2-8}$ and $F_{\mathrm{ROH}8}$, respectively. An ROH of less than 8 Mb is formed more than six generations ago in the theory (Howrigan et al. 2011) and in actual subpopulations of Angus cattle (Cardoso et al. 2020). Several reports have indicated that inbreeding coefficients based on ROH2–8 and ROH8 have different effects on reproductive traits (Makanjuola, Maltecca, et al. 2020) and growth traits (Lozada‐Soto et al. 2021) in cattle. Therefore, this study estimated genomic inbreeding coefficients using two lengths ($F_{\mathrm{ROH}2-8}$ and $F_{\mathrm{ROH}8}$) and the entire length ($F_{\mathrm{ROH}2}$) of the ROH.

$F_{\mathrm{HBD}}$ is based on homozygous‐by‐descent (HBD) segments, which are chromosomal segments inherited twice from a common ancestor without recombination. HBD segments result in long stretches of homozygous genotypes (i.e., ROH), and the ROH segment is autozygous. The classification of HBD segments was estimated using the *RZooRoH* package in the R software (Bertrand et al. 2019), which is a model‐based approach based on a hidden Markov model (HMM) to identify HBD segments (Druet and Gautier 2017). In this model, the genomic region is described as a mosaic of HBD and non‐HBD segments with an HMM. The age of inbreeding is estimated for HBD classes based on the transition probability between different HBD and non‐HBD segments and is conditional on class specificity. The probability of remaining in a particular state is calculated as $e^{-R_{k}}$, where $R_{k}$ is the rate specific to the *k*th class. This implies that the length of an HBD segment of any class follows an exponential distribution with rate $R_{k}$. In this study, 10 HBD classes (default settings) were used, and the proportion of HBD loci to all loci was calculated and defined as $F_{\mathrm{HBD}}$. To calculate $F_{\mathrm{HBD}}$, SNPs were assessed using the exclusion criterion of MAF < 0.01.

### 2.3 Estimation of Genome‐Wide Inbreeding Depression

ASReml 4.2 software (Gilmour et al. 2021) was used to estimate the effects of genome‐wide inbreeding on semen production traits, using the following model:

(1) y=Xb+Zu+Wpe+βf+e,

where **y** is a vector of phenotypic value; **X**, **Z**, and **W** are the incidence matrices; **b** is a vector of fixed effects due to contemporary group comprising the year‐season‐AI center (408 levels in JB and 371 levels in HOL) and collection interval (five levels: ≤ 3, 4, 5, 6, and ≥ 7 days) and covariate for bull age (linear and quadratic), and **u** is a vector of breeding value ($u\sim N(0,\sigma_{u}^{2}A$)), where $\sigma_{u}^{2}$ is the additive genetic variance; and **A** is the additive relationship matrix. **pe** is a vector of permanent environmental effects ($\mathrm{pe}\sim N\left(0,\sigma_{\mathrm{pe}}^{2}I\right)$), where $\sigma_{\mathrm{pe}}^{2}$ is the permanent environmental variance, and **I** is the identity matrix. **e** is a vector of error effects ($e\sim N\left(0,\sigma_{e}^{2}I\right)$), where $\sigma_{e}^{2}$ is the error variance. β is the regression coefficient of semen production traits on inbreeding coefficients, and **f** is a vector of inbreeding coefficients. When estimating the effects of $F_{\mathrm{ROH}2-8}$ and $F_{\mathrm{ROH}8}$, we fitted simultaneously these two effects, where we assumed $\beta_{\mathrm{ROH}2-8}f_{\mathrm{ROH}2-8}+\beta_{\mathrm{ROH}8}f_{\mathrm{ROH}8}$ instead of βf. $\beta_{\mathrm{ROH}2-8}$ and $\beta_{\mathrm{ROH}8}$ are the regression coefficients of $F_{\mathrm{ROH}2-8}$ and $F_{\mathrm{ROH}8}$, respectively, and $f_{\mathrm{ROH}2-8}$ and $f_{\mathrm{ROH}8}$ are vectors of $F_{\mathrm{ROH}2-8}$ and $F_{\mathrm{ROH}8}$, respectively. The *z*‐statistic was calculated as $\hat{\beta }/\mathrm{se}\left(\hat{\beta }\right)$, where $\hat{\beta }$ is the estimated regression coefficient on the inbreeding coefficient using Model (1) and $\mathrm{se}\left(\hat{\beta }\right)$ is the standard error (SE) of $\hat{\beta }$. The significance of the regression coefficients was tested using a *z*‐test based on the *z*‐statistic.

### 2.4 Mapping of Genomic Regions Associated With Inbreeding Depression

To map homozygous regions within the genome associated with semen production traits, ROH‐based GWAS was designed to investigate the effects of ROH across the genome on semen production traits (Ferenčaković et al. 2017; Ghoreishifar et al. 2023; Pryce et al. 2014). First, the adjusted phenotypic values of animal *i* ($y_{\mathrm{adj},i}$) were derived from Model (1) without inbreeding effects (βf) and calculated by

$$y_{\mathrm{adj},i}=\hat{u_{i}}+\hat{{\mathrm{pe}}_{i}}+\text{mean}\left(\hat{e_{\mathrm{ij}}}\right),$$

where $\hat{u_{i}}$ is the predicted breeding value of animal *i*, $\hat{{\mathrm{pe}}_{i}}$ is the predicted permanent environmental effect of animal *i*, and $\text{mean}\left(\hat{e_{\mathrm{ij}}}\right)$ is the mean error of the *j*th record of animal *i*.

Second, the adjusted phenotypic values were used as dependent traits in a linear mixed model implemented in ASReml 4.2 software (Gilmour et al. 2021), with each SNP fitted individually using the following model:

$$y_{\mathrm{adj}}=\mu 1_{n}+\alpha_{k}x_{k}+\beta_{k}h_{k}+Zu+\varepsilon ,$$

where $y_{\mathrm{adj}}$ is a vector of the adjusted phenotypic values; *μ* is the mean; $1_{n}$ is a vector of *n* ones; **Z** is the incidence matrix; **u** is a vector of breeding value ($u\sim N(0,\sigma_{u}^{2}A$)), where $\sigma_{u}^{2}$ is the additive genetic variance; **A** is the additive relationship matrix; ε is a vector of residuals ($\varepsilon \sim N\left(0,\sigma_{\varepsilon }^{2}I\right)$), where $\sigma_{\varepsilon }^{2}$ is the residual variance; $\alpha_{k}$ is the allele substitute effect at the *k*th SNP, $x_{k}$ is a vector of SNP genotypes at the *k*th SNP, $\beta_{k}$ is the regression coefficient on the ROH state at the *k*th SNP, and $h_{k}$ is a vector of ROH state at the *k*th SNP. The ROH state of the *k*th SNP on the bull was coded as 1 when the SNP was present within an ROH and 0 otherwise. In this study, two ROH lengths (i.e., ROH2–8 and ROH8) were assessed for inbreeding depression in genomic regions. The effect of ROH was estimated at each position by simultaneously correcting for the additive effect of the SNP. The *z*‐statistic of the ROH effect for each SNP was calculated in the same manner as the estimation of genome‐wide inbreeding depression to test the significance of the regression coefficients.

Bonferroni correction was applied to determine the genome‐wide significance thresholds as *p* = 0.05/nSNP, where nSNP is the number of SNPs used for GWAS. In addition, the genome‐wide suggestive threshold was defined as *p* = 1/nSNP (Lander and Kruglyak 1995). A total of 12,648 and 36,584 SNPs in JB and 27,150 and 36,705 SNPs in HOL were extracted from ROH2–8 and ROH8, respectively, and used for the GWAS. These extractions were conducted under the condition that the number of bulls with an ROH state coded as 1 was greater than 20 for each SNP.

## 3 Results

### 3.1 Estimation of Genome‐Wide Inbreeding Depression

**FIGURE 1:**
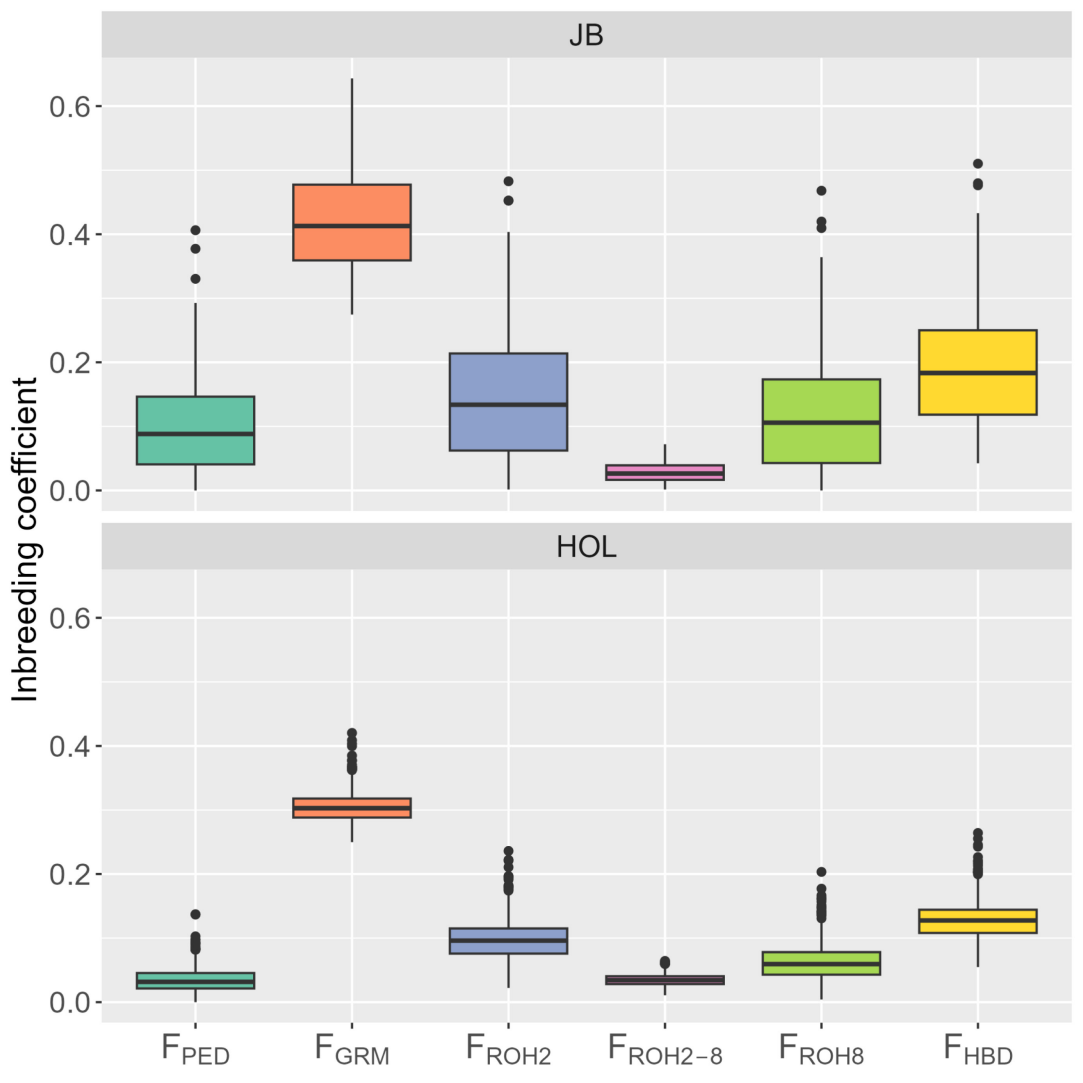
Box plots of six measures of inbreeding coefficient in Japanese Black bulls (JB) and Holstein bulls (HOL). $F_{\mathrm{PED}}$ = pedigree‐based inbreeding coefficient, $F_{\mathrm{GRM}}$ = genomic inbreeding coefficient from genomic relationship matrix, $F_{\mathrm{ROH}2}$ = genomic inbreeding coefficient from runs of homozygosity (ROH) of 2 Mb or larger in length, $F_{\mathrm{ROH}2-8}$ = genomic inbreeding coefficient from ROH of 2–8 Mb in length, $F_{\mathrm{ROH}8}$ = genomic inbreeding coefficient from ROH of 8 Mb or larger in length, $F_{\mathrm{HBD}}$ = genomic inbreeding coefficient from model‐based homozygous‐by‐descent (HBD) segments.

One pedigree‐based and five genomic inbreeding coefficients were calculated for JB and HOL (Figure 1). The mean ± SD of inbreeding coefficients were 0.10 ± 0.07 and 0.03 ± 0.02 for $F_{\mathrm{PED}}$, 0.42 ± 0.07 and 0.30 ± 0.02 for $F_{\mathrm{GRM}}$, 0.14 ± 0.09 and 0.10 ± 0.03 for $F_{\mathrm{ROH}2}$, and 0.19 ± 0.08 and 0.13 ± 0.03 for $F_{\mathrm{HBD}}$ in JB and HOL, respectively. The values of each inbreeding coefficient showed a similar trend between breeds, but each inbreeding coefficient of JB showed greater variation compared to those of HOL. Specifically, $F_{\mathrm{GRM}}$ exhibited the highest value, followed by $F_{\mathrm{HBD}}$, $F_{\mathrm{ROH}2}$, and $F_{\mathrm{PED}}$ in descending order. Furthermore, the inbreeding coefficient calculated using ROH showed that $F_{\mathrm{ROH}8}$ (0.11 ± 0.08 in JB and 0.06 ± 0.03 in HOL) showed a higher value than $F_{\mathrm{ROH}2-8}$ (0.03 ± 0.01 in JB and 0.03 ± 0.01 in HOL).

The regression coefficients of semen production traits on inbreeding coefficients were estimated for JB and HOL and are shown in Table 1. No significant inbreeding effect on VOL was observed in either breed. Except for $F_{\mathrm{ROH}2-8}$, significant negative effects of inbreeding on four semen production traits (NUM, MOT, aMOT, and CON) were observed in JB, whereas only NUM was significantly affected in HOL.

**TABLE 1:** Estimated regression coefficients of semen production traits on inbreeding coefficients in Japanese Black and Holstein bulls.

Traits	Inbreeding coefficients^a^	Japanese Black			Holstein		
VOL	*F*_PED_	−0.10	(0.13)		−0.16	(0.13)	
	*F*_GRM_	−0.30	(0.23)		−0.17	(0.15)	
	*F*_ROH2_	−0.17	(0.15)		−0.20	(0.17)	
	*F*_ROH2–8_	−0.15	(0.22)		0.03	(0.18)	
	*F*_ROH8_	−0.10	(0.15)		−0.18	(0.13)	
	*F*_HBD_	−0.26	(0.16)		−0.24	(0.17)	
NUM	*F*_PED_	−4.98	(1.39)	***	−3.38	(1.69)	*
	*F*_GRM_	−9.89	(2.44)	***	−4.31	(1.95)	*
	*F*_ROH2_	−6.44	(1.61)	***	−4.68	(2.14)	*
	*F*_ROH2–8_	0.29	(2.33)		−1.65	(2.35)	
	*F*_ROH8_	−6.06	(1.61)	***	−3.59	(1.75)	*
	*F*_HBD_	−7.35	(1.72)	***	−5.20	(2.16)	*
CON	*F*_PED_	−0.40	(0.17)	*	−0.20	(0.19)	
	*F*_GRM_	−0.76	(0.30)	*	−0.24	(0.22)	
	*F*_ROH2_	−0.51	(0.20)	**	−0.20	(0.24)	
	*F*_ROH2–8_	0.39	(0.29)		−0.05	(0.26)	
	*F*_ROH8_	−0.61	(0.20)	**	−0.16	(0.20)	
	*F*_HBD_	−0.50	(0.21)	*	−0.26	(0.24)	
MOT	*F*_PED_	−0.86	(0.16)	***	−0.16	(1.22)	
	*F*_GRM_	−1.47	(0.28)	***	−0.43	(0.26)	
	*F*_ROH2_	−1.03	(0.19)	***	−0.48	(0.28)	
	*F*_ROH2–8_	0.24	(0.27)		−0.21	(0.31)	
	*F*_ROH8_	−1.04	(0.18)	***	−0.36	(0.23)	
	*F*_HBD_	−1.11	(0.20)	***	−0.37	(0.29)	
aMOT	*F*_PED_	−0.72	(0.23)	**	−0.15	(0.26)	
	*F*_GRM_	−1.56	(0.40)	***	−0.22	(0.30)	
	*F*_ROH2_	−1.17	(0.26)	***	−0.25	(0.33)	
	*F*_ROH2–8_	0.15	(0.38)		−0.02	(0.37)	
	*F*_ROH8_	−1.13	(0.26)	***	−0.20	(0.27)	
	*F*_HBD_	−1.22	(0.28)	***	−0.14	(0.34)	

Abbreviations: aMOT, sperm motility after freeze–thawing; CON, sperm concentration; MOT, sperm motility; NUM, sperm number; VOL, semen volume.

^a^ *F*_GRM_ = genomic inbreeding coefficient from the genomic relationship matrix; *F*_HBD_ = genomic inbreeding coefficient from model‐based homozygous‐by‐descent (HBD) segments; *F*_PED_ = pedigree‐based inbreeding coefficient; *F*_ROH2_ = genomic inbreeding coefficient from runs of homozygosity (ROH) of 2 Mb or larger in length; *F*_ROH2–8_ = genomic inbreeding coefficient from ROH of 2–8 Mb in length; *F*_ROH8_ = genomic inbreeding coefficient from ROH of 8 Mb or larger in length. Statistical significance is indicated by *, **, and *** for *p* < 0.05, *p* < 0.01, and *p* < 0.001, respectively. Standard errors are indicated in parentheses.

### 3.2 Mapping of Genomic Regions Associated With Inbreeding Depression

**FIGURE 2:**
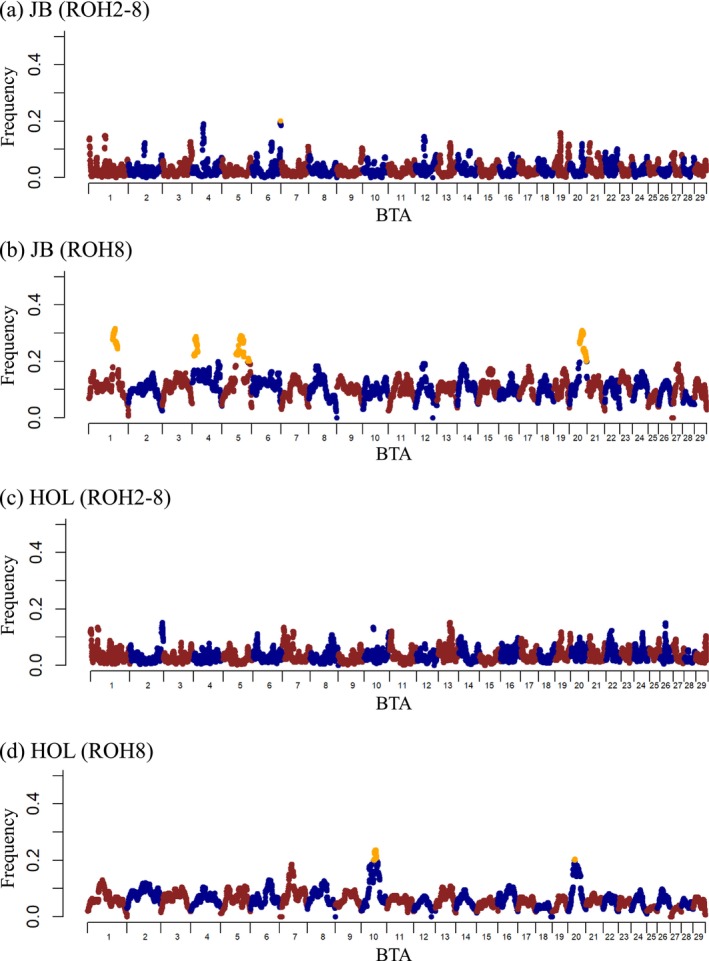
Genome‐wide plots of the distribution of runs of homozygosity (ROH) islands in the genomes of Japanese Black bulls (JB) and Holstein bulls (HOL) under different ROH definitions. (a) JB's genome with ROH length of 2–8 Mb (ROH2–8). (b) JB's genome with ROH length exceeding 8 Mb (ROH8). (c) HOL's genome with ROH2–8. (d) HOL's genome with ROH8. The *x*‐axis represents *Bos taurus* autosome (BTA) number, and the *y*‐axis shows frequency (%) of overlapping ROH shared among individuals. Orange dots represent the frequency of overlapping ROH over 0.2.

Figure 2 shows a genome‐wide plot of the distribution of ROH islands in JB and HOL under two distinct ROH lengths (ROH2–8 and ROH8). For ROH2–8 in JB, BTA 6 had one region with a frequency of ROH over 0.2. For ROH8 in JB, BTA 1, 4, 5, and 20 had regions with a frequency of ROH over 0.2. For ROH2–8 in the HOL, no region had a frequency of ROH > 0.2. For ROH8, BTA 10 and 20 had regions with a frequency of ROH > 0.2. For the ROH regions with a frequency of ROH > 0.2, no common regions were observed between JB and HOL.

**FIGURE 3:**
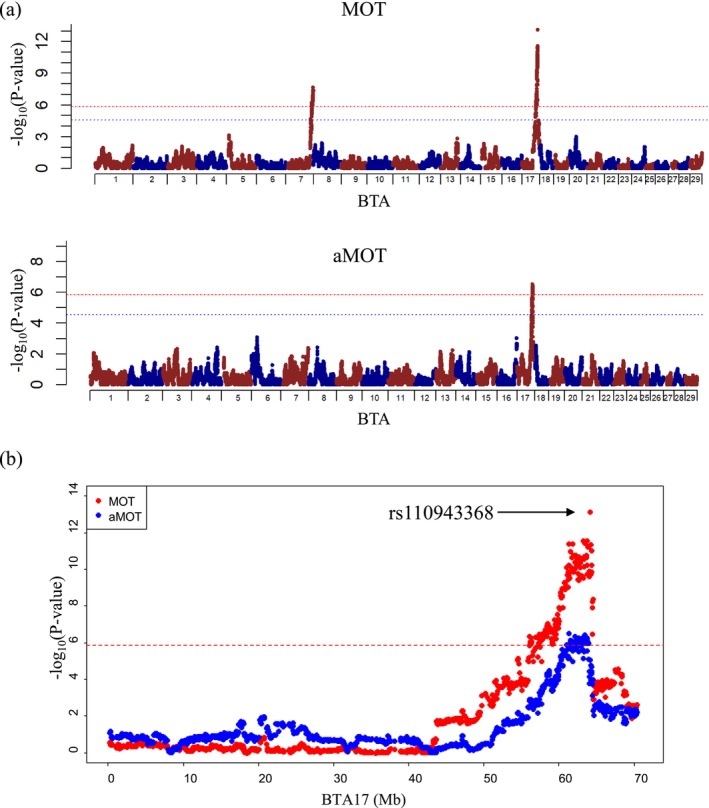
Genome‐wide plots and significant regions for sperm motility (MOT) and MOT after freeze–thawing (aMOT) in Holstein bulls. (a) Genome‐wide plots and (b) regional plots on BTA 17 (0–74 Mb) representing genome‐wide significant association with MOT and aMOT. Results of runs of homozygosity (ROH) of lengths over 8 Mb. The *x*‐axis indicates *Bos taurus* autosome (BTA) number, and the *y*‐axis indicates *p* values (−log_10_). Horizontal red and blue lines represent genome‐wide significant and suggestive thresholds, respectively.

To assess the impact of ROH on semen production traits per SNP, genome‐wide and regional plots of *p* values with genome‐wide significant SNPs in HOL are shown in Figures 3a and 3b, respectively. A summary of the significant regions in JB and HOL is shown in Table 2. Genome‐wide plots of *p* values with genome‐wide suggestive SNPs in JB and HOL are shown in Figures S1 and S2, respectively. A summary of the suggestive regions is shown in Table 2. For ROH2–8, there were no significant genome‐wide SNPs in either breed, and SNPs in BTA 14 in JB and BTA 2 in HOL were associated with CON and MOT, respectively. For ROH8, there were no significant genome‐wide SNPs in JB; however, SNPs on BTA 2, 4, 9, 14, 19, and 23 were suggestively associated with MOT and aMOT. The SNPs in BTA 7 and 17 were significantly associated with MOT and aMOT in HOL. The significant region on BTA 17 (from 61 to 64 Mb) overlapped for MOT and aMOT in HOL (Figure 3b), and the rs110943368 SNP had the highest significant effect on MOT with *p* = $7.4\times 10^{-14}$. The rs43296066 SNP on BTA 7 was the second most significant region for MOT, with a *p* = $2.1\times 10^{-8}$. Furthermore, the suggestive and significant regions identified in both breeds did not coincide with the areas of high ROH frequency (see also Figure 2).

**TABLE 2:** Summary of regions significantly associated with semen production traits by genome‐wide association studies in Japanese Black and Holstein bulls.

	The length of ROH in analysis^a^		Position (bp)		refSNP ID				
Traits		BTA	Start	End	Start	End	nSNP	Min *p* ^b^	
Japanese Black									
CON	ROH2–8	14	26,068,656	26,691,608	rs41666770	rs41579040	15	7.5 × 10^−6^	*
MOT	ROH8	4	59,730,602	66,948,425	rs41624198	rs110315476	9	9.3 × 10^−6^	*
		4	103,706,383	115,153,844	rs41573224	rs42737300	36	4.0 × 10^−6^	*
		9	13,684,115	13,809,560	rs43130416	rs42758714	4	1.8 × 10^−5^	*
		9	69,514,180		rs109780456		1	2.7 × 10^−5^	*
		14	11,763,629		rs109276546		1	1.1 × 10^−5^	*
		19	19,065,681	26,091,138	rs41579814	rs41619528	78	4.8 × 10^−6^	*
		23	33,880,496	34,750,192	rs110142633	rs110520713	7	5.7 × 10^−6^	*
aMOT	ROH8	2	54,364,683	55,101,502	rs41588086	rs110504727	14	9.9 × 10^−6^	*
		2	126,602,788	127,326,815	rs109867203	rs109916584	14	1.1 × 10^−5^	*
		4	62,333,882	66,948,425	rs29017015	rs110315476	14	4.4 × 10^−6^	*
		4	104,487,046	105,439,327	rs110534108	rs109583025	3	1.9 × 10^−5^	*
Holstein									
MOT	ROH2–8	2	94,626,674		rs41641871		1	2.3 × 10^−5^	*
	ROH8	7	104,401,635	110,478,001	rs43531510	rs42773948	118	2.1 × 10^−8^	**
		17	56,009,167	64,477,583	rs42262925	rs109048924	148	7.4 × 10^−14^	**
aMOT	ROH8	17	60,983,050	63,860,049	rs110263418	rs110266660	38	3.1 × 10^−7^	**

Abbreviations: aMOT, sperm motility after freeze–thawing; BTA: *Bos taurus* autosome; CON, sperm concentration; MOT, sperm motility; nSNP: number of significant or suggestive SNPs within the detected region; refSNP ID: single‐nucleotide polymorphism (SNP) identification cataloged in the National Center for Biotechnology Information SNP database.

^a^ Runs of homozygosity (ROH) of 2–8 Mb in length (ROH2–8) and ROH of 8 Mb or larger in length (ROH8).

^b^ Min *p*: minimum *p* value within the detected region. Statistical significance was labeled as * and ** for genome‐wide 5% suggestive and significance levels, respectively.

**FIGURE 4:**
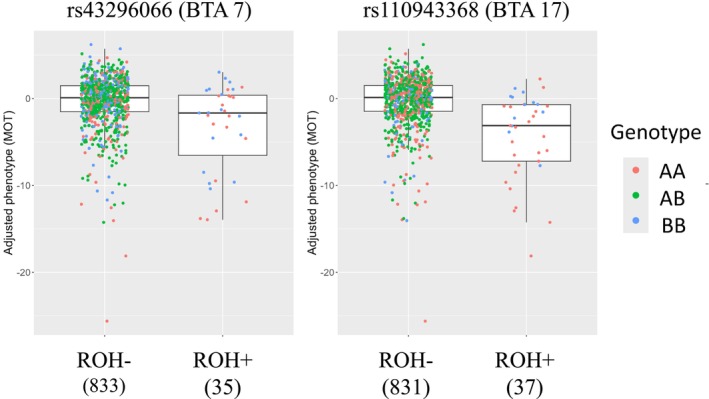
Box plots representing the relationship between run of homozygosity (ROH) states of two genome‐wide significant single‐nucleotide polymorphisms (SNPs) (rs43296066 and rs110943368) and sperm motility (MOT) in Holstein bulls. ROH state of SNP on bull was represented as ROH+ when SNP is present within an ROH and represented as ROH− otherwise. rs43296066 and rs110943368 SNPs are located on *Bos taurus* autosome (BTA) 7 and 17, respectively; the *y*‐axis indicates the adjusted phenotypic values of MOT. The bold line indicates the median of the traits per SNP, and each dot represents a unique animal, and different colors show different genotypes. In total, 868 Holstein bulls were used, and the number of bulls per ROH state is shown in parentheses.

With regard to the impact of ROH8 on MOT, the distributions of adjusted phenotypic values for each ROH state at rs43296066 on BTA 7 and rs110943368 SNP on BTA 17 in HOL are shown in Figure 4. ROH state of the SNP on bull was represented as ROH+ when the SNP was present within an ROH and represented as ROH− otherwise. The bulls with ROH+ had lower phenotypic values of MOT than those with ROH− in both SNPs. At rs110943368 SNP, bulls with AA genotype had lower phenotypic values than those with BB genotype (mean ± SD were −5.6 ± 5.1 and 1.5 ± 2.4 for AA and BB genotypes, respectively). In contrast, at rs43296066 SNP, the difference of phenotypic values in between bulls with AA and BB genotypes was lower than that at rs110943368 SNP (mean ± SD were −4.2 ± 5.5 and −2.5 ± 4.5 for AA and BB genotypes, respectively).

## 4 Discussion

### 4.1 Estimation of Genome‐Wide Inbreeding Depression

In this study, we hypothesized that genome‐wide inbreeding negatively affects semen production traits. Our results indicated that pedigree‐based and genomic inbreeding coefficients exhibited a significantly negative association with NUM, CON, MOT, and aMOT in JB and NUM in HOL. The results indicated that the direction of the effect of inbreeding on semen production traits was consistent across breeds. Similar results were shown in Atagi et al. (2017), who reported negative inbreeding effects on semen production traits in JB and HOL using pedigree‐based inbreeding coefficient. This result is also consistent with the findings of Ferenčaković et al. (2017), who reported that genome‐wide inbreeding has a negative effect on semen production traits in Austrian Fleckvieh bulls. In contrast, the traits affected by inbreeding depression differed between breeds. One possible explanation for these results is that the distribution of inbreeding coefficients, excluding $F_{\mathrm{ROH}2-8}$, differed between JB and HOL, with JB exhibiting a larger distribution range. Japanese Black cattle have unique genetic characteristics, with *N*_e_ of approximately 20 (Nomura et al. 2001). This can be attributed to the fact that a few sires with high estimated breeding value for marbling have been utilized multiple times across generations. In contrast, Holstein cattle are reared worldwide, and their *N*_e_ is higher than that of Japanese Black cattle (Makanjuola, Miglior, et al. 2020). Consequently, it is plausible that the differences in *N*_e_ between the two breeds may have influenced the differences in the distribution of inbreeding coefficients between them. However, as the SNP array used in this study was not developed by extracting polymorphic SNPs from the Japanese Black cattle, caution should be exercised when comparing inbreeding coefficients between breeds.

In this study, we calculated pedigree‐based and genomic inbreeding coefficients based on GRM and homozygous segments and investigated the effect of inbreeding depression on semen production traits in cattle. No large differences were observed in the results among $F_{\mathrm{PED}}$, $F_{\mathrm{GRM}}$, $F_{\mathrm{ROH}2}$, and $F_{\mathrm{HBD}}$ in either breed in terms of significance test, even if the pedigree was traced back to only four generations and was used for $F_{\mathrm{PED}}$ calculation. Some studies have demonstrated that $F_{\mathrm{GRM}}$, $F_{\mathrm{ROH}2}$, and $F_{\mathrm{HBD}}$ are effective estimators of the effects of inbreeding on production and reproductive traits in cattle populations (Bjelland et al. 2013; Doekes et al. 2019; Lozada‐Soto et al. 2024; Nishio et al. 2023). Therefore, these three measures are recommended for estimating genome‐wide inbreeding depression in cattle. However, it should be noted that this study evaluated the significance of regression coefficients and their signs. In this study, $F_{\mathrm{GRM}}$ showed higher values and higher rates of inbreeding depression than those of other coefficients. This discrepancy is attributable to the varying ranges of inbreeding coefficients between $F_{\mathrm{GRM}}$ and other coefficients. The $F_{\mathrm{GRM}}$ is based on the assumption that an allele frequency at the base population is fixed at 0.5 in all loci, resulting in a range of values from −1 to 1 (Villanueva et al. 2021). In contrast, the range of other coefficients was from 0 to 1. Consequently, comparing estimates between $F_{\mathrm{GRM}}$ and other coefficients is difficult, and the significance of regression coefficients and their signs were evaluated.

ROH‐based genomic inbreeding coefficients account for age of inbreeding (Ceballos et al. 2018; Keller et al. 2011). In this study, we calculated ROH‐based genomic inbreeding coefficients using two different lengths of ROHs (ROH2–8 and ROH8) and investigated the inbreeding depression of semen production traits in cattle. Our results showed no effect of $F_{\mathrm{ROH}2-8}$ on semen production traits; however, negative effect of $F_{\mathrm{ROH}2}$ and $F_{\mathrm{ROH}8}$ on semen production traits were observed in both JB and HOL. Because the values of $F_{\mathrm{ROH}8}$ exhibited a larger SD than those of $F_{\mathrm{ROH}2-8}$, it can be concluded that most of the variation in $F_{\mathrm{ROH}2}$ can be explained by those of $F_{\mathrm{ROH}8}$ in our population. In general, shorter ROHs reflect more ancient inbreeding resulting from a common ancestor traced back to multiple generations within the pedigree. Conversely, longer ROHs reflect inbreeding that occurred in recent generations (Ceballos et al. 2018; Keller et al. 2011). The length of the autozygous segment was expected to follow an exponential distribution with a mean of 1/(2*G*) Morgans, where *G* is the number of generations since its common ancestor (Howrigan et al. 2011). Consequently, the number of generations from selection events can be inferred from ROH length and frequency. Mutations in the long ROH are expected to be more harmful than those in the short ROH, because there have been fewer generations for genetic purging and selection against harmful recessive alleles (Hedrick and Garcia‐Dorado 2016). Therefore, a long ROH may be associated with greater inbreeding depression than a short ROH, and our results support this hypothesis. In addition, some previous studies also reported that, in contrast to short ROH, long ROH are negatively associated with semen production traits in HOL (Ghoreishifar et al. 2023), performance and female reproductive traits in Holstein cows (Makanjuola, Maltecca, et al. 2020), and growth traits in Angus cattle (Lozada‐Soto et al. 2021). These results indicate the importance of using long‐range ROH information to calculate genomic inbreeding coefficients when evaluating genome‐wide inbreeding depression.

### 4.2 Mapping of Genomic Regions Associated With Inbreeding Depression

The use of ROH information to evaluate inbreeding offers several advantages. This approach facilitates not only the estimation of genome‐wide inbreeding but also the identification of genomic regions underlying inbreeding depression (Ferenčaković et al. 2017; Ghoreishifar et al. 2023; Pryce et al. 2014; Scott et al. 2025). Consequently, we performed an ROH‐based GWAS for semen production traits, accounting for short ROH (ROH2–8) and long ROH (ROH8). The results for ROH2–8 showed that only one genome‐wide suggestive region was detected in each breed. In contrast, the results of ROH8 indicated that many genome‐wide suggestive SNPs were detected in JB, and genome‐wide significant SNPs were detected on BTA 7 and 17 for MOT and on BTA 17 for aMOT in HOL. Therefore, our results indicate that some genomic regions affected inbreeding depression on semen production traits in both breeds, but the effects of age of inbreeding were different between JB and HOL. Some studies have mapped the genomic regions contributing to inbreeding depression in cattle populations (Ferenčaković et al. 2017; Ghoreishifar et al. 2023; Pryce et al. 2014; Scott et al. 2025). However, no studies have mapped genomic regions using separate analyses of long and short ROH. This study indicated that more regions exhibiting inbreeding depression were observed in the long ROHs than in the short ROHs, which is consistent with the results of genome‐wide inbreeding studies. Therefore, considering the length of the ROH when mapping genomic regions associated with inbreeding depression may lead to a deeper understanding of the mechanisms underlying inbreeding depression.

For the significant region of BTA 17 identified in HOL, the most significant SNP was rs110943368, located at 64,109,516 bp. The SNP was located near the rs41843851 SNP at 64,011,119 bp and was included in the SNP array used in this study. In a previous study, we performed a GWAS that accounted for nonadditive effects using the same population as in the present study (Nagai et al. 2022a). We found that rs41843851 exhibited the most significant nonadditive effects on semen production traits, including MOT and aMOT, in HOL. Consequently, the rs41843851 SNP is considered to have nonadditive effects on the semen production traits. In addition, the rs41843851 SNP was the same as a significant nonadditive SNP for sire conception rates in Holstein cattle (Nicolini et al. 2018). In this population, bulls with the BB genotype had lower phenotypic values for semen production traits than those with the AA and AB genotypes for the rs41843851 SNP. Among the 28 bulls with the BB genotype at rs41843851, 20 bulls had ROH+ on ROH8 at the same SNP. These results indicated that this region was thought to have been homozygous by recent inbreeding, suggesting the possibility of a causal recessive mutation near this SNP. The *ubiquitin‐specific protease 30* (USP30) and *transmembrane protein 119* (TMEM119) genes were located within this region. USP30 is a deubiquitinating enzyme embedded in the mitochondrial outer membrane that regulates mitochondrial morphology (Nakamura and Hirose 2008) and is expressed in cattle testes. The USP family is associated with male infertility in humans (Bedard et al. 2011; Lee et al. 2003; Paduch et al. 2005). TMEM119 is a single transmembrane protein expressed in spermatocytes and spermatids, and TMEM119 knockout mice showed decreased testis weight and sperm number (Mizuhashi et al. 2015). Consequently, the significant region identified by the ROH‐based GWAS suggests the possibility of containing a harmful recessive mutation in semen production traits. Furthermore, our study demonstrates the efficacy of ROH‐based GWAS in identifying causal recessive mutations that contribute to inbreeding depression.

In this study, the rs43296066 SNP in BTA 7 was identified as the second most significant SNP associated with MOT in HOL. Nagai et al. (2022a) conducted a GWAS that accounted for nonadditive effects in this population; however, we did not detect any significant SNPs in this region. The difference of phenotypic values between bulls with AA and BB genotypes at the rs43296066 SNP was lower than that at the rs110943368 SNP (see Figure 4). Consequently, although a causal recessive mutation may exist in this region, it is suggested that this mutation may not be in linkage disequilibrium with the SNPs on the SNP array used in this study. As candidate genes within this region, *mannosidase alpha class 2A member 1* (*MAN2A1*) gene and *transmembrane protein 232* (*TMEM232*) gene were located within the region. *MAN2A1* encodes a glycosyl hydrolase that localizes to the Golgi apparatus and catalyzes the final hydrolytic step of the asparagine‐linked oligosaccharide (N‐glycan) maturation pathway (https://www.ncbi.nlm.nih.gov/gene/4124). This gene is associated with scrotal circumference in cattle (Dementshuk et al. 2018). *TMEM232* encodes a transmembrane protein that is predicted to be involved in flagellated sperm motility and spermatid cytoplasm removal during the spermiation of flagellated sperm (https://www.ncbi.nlm.nih.gov/gene/642987). This gene is involved in sperm flagellum formation in mice (Cai et al. 2024). Given that some candidate genes have been reported within this region, ROH‐based GWAS can detect genomic regions associated with inbreeding depression that cannot be detected by GWAS alone, thereby accounting for nonadditive effects. Further research is necessary to investigate the association between this region and semen production traits.

### 4.3 Evaluation of Inbreeding Depression Using Genome‐Wide and Regional Approaches

This study estimated the genome‐wide inbreeding depression and mapped the genomic regions associated with inbreeding depression for semen production traits in two different breeds. Our results indicate that genome‐wide inbreeding significantly affects most semen production traits in JB. However, no significant regions associated with the inbreeding depression of semen production traits were detected, although some suggestive regions were identified. These results indicate that many small SNPs may influence the inbreeding depression of semen production traits in JB. In contrast, a significant effect of genome‐wide inbreeding only on NUM was estimated in HOL, whereas two significant regions were identified in MOT and aMOT. These results indicate that only a few SNPs with large effects may have an impact on the inbreeding depression of semen production traits in HOL. Accordingly, the present study revealed that the effects of inbreeding were either regional (a small number of SNPs with large effects) or genome‐wide (many SNPs with small effects), and these effects varied among the target populations. Several studies evaluated inbreeding depression using both genome‐wide and regional approaches in cattle populations and showed the different effects of inbreeding among populations (Ferenčaković et al. 2017; Ghoreishifar et al. 2023; Pryce et al. 2014; Scott et al. 2025). Therefore, it is important to investigate the use of both approaches to evaluate inbreeding depression. In this case, the approach using the long length of ROHs appears useful and effective both for estimating genome‐wide inbreeding levels and mapping inbreeding effects at the level of individual SNPs.

To effectively manage genetic selection in cattle populations by accounting for inbreeding depression, it is essential to consider differences in the genetic backgrounds of the target population. If many SNPs with small effects affect inbreeding depression, such as JB in this study, genomic inbreeding coefficients could be useful for controlling inbreeding while improving target traits. For example, optimal contribution selection (Meuwissen 1997) is a method of genetic improvement that restricts the rate of inbreeding. Genomic inbreeding coefficients can be applied to this method (Wellmann 2019), and some studies have utilized this method in simulation analyses before applying it to real cattle populations (Gautason et al. 2023; Kohl et al. 2020). However, if a few SNPs with large effects affect inbreeding depression, such as HOL, marker‐assisted selection by excluding recessive harmful alleles in the target population could be useful for improving target traits. For example, pairs of bulls and cows that do not share ROHs+ in BTA 7 and 17 could have been selected in our case if a recessive harmful mutation had not been identified. As there are several methods for controlling inbreeding depression, it is important to understand the genetic basis of inbreeding depression in the target population using both approaches.

This study estimated the genome‐wide inbreeding depression and mapped the genomic regions associated with inbreeding depression for semen production traits in beef and dairy breeds. Our results indicate the importance of utilizing long ROH information when evaluating genomic inbreeding depression, both regionally and across the genome, in both breeds. Genome‐wide inbreeding depression was observed in NUM, MOT, aMOT, and CON in JB but only in NUM in HOL. However, significant regional effects were not observed in JB but were observed in MOT and aMOT in HOL. Therefore, this study revealed that the effects of inbreeding were either regional (a small number of SNPs with large effects) or genome‐wide (many SNPs with small effects), and these effects varied among the target populations. Consequently, it is important to examine both approaches when evaluating inbreeding depression in cattle. In this case, the approach using long ROHs appears useful and effective both for estimating genome‐wide inbreeding levels and mapping inbreeding effects at the level of individual SNPs.

## Funding

This study was partially supported by the JST SPRING (grant number JPMJSP2124).

## Ethics Statement

All phenotypic and SNP data were recorded for routine beef and dairy cattle management, following the Japanese rules and regulations for animal care. Approval from the Animal Care and Use Committee was not required for this study because the data were obtained from an existing database of the LIAJ and the HCAJ.

## Conflicts of Interest

The authors declare no conflicts of interest.

## Supporting information


**Figure S1:** Genome‐wide plots representing genome‐wide suggestive association with (a) sperm motility (MOT), (b) MOT after freeze–thawing (aMOT), and (c) sperm concentration (CON) in Japanese Black bulls. Results of runs of homozygosity (ROH) of lengths of 2–8 Mb (ROH2‐8) and > 8 Mb (ROH8); the *x*‐axis indicates *Bos taurus* autosome (BTA) number, and the *y*‐axis indicates *p* values (−log_10_). Horizontal red and blue lines represent genome‐wide significant and suggestive thresholds, respectively.

**Figure S2:** Genome‐wide plots representing genome‐wide suggestive association with (a) sperm motility (MOT) in Holstein bulls. Results of runs of homozygosity (ROH) of lengths of 2–8 Mb (ROH2–8); the *x*‐axis indicates *Bos taurus* autosome (BTA) number, and the *y*‐axis indicates *p* values (−log_10_). Horizontal red and blue lines represent the genome‐wide significant and suggestive thresholds, respectively.

**Table S1:** Descriptive statistics of semen production traits in Japanese Black and Holstein bulls.
