# Supplementary material for: Impact of Genome‐Wide and Regional Inbreeding on Semen Production Traits in Beef and Dairy Bulls
Source: Anim Sci J. 2025 Dec 3;96(1):e70138. doi: 10.1111/asj.70138 (PMC12675868; doi:10.1111/asj.70138)
Supplement: Supplementary file 1 — Figure S1: Genome‐wide plots representing genome‐wide suggestive association with (a) sperm motility (MOT), (b) MOT after freeze–thawing (aMOT), and (c) sperm concentration (CON) in Japanese Black bulls. Results of runs of homozygosity (ROH) of lengths of 2–8 Mb (ROH2‐8) and > 8 Mb (ROH8); the x‐axis indicates Bos taurus autosome (BTA) number, and the y‐axis indicates p values (−log10). Horizontal red and blue lines represent genome‐wide significant and suggestive thresholds, respectively. [file ASJ-96-e70138-s002.pdf]

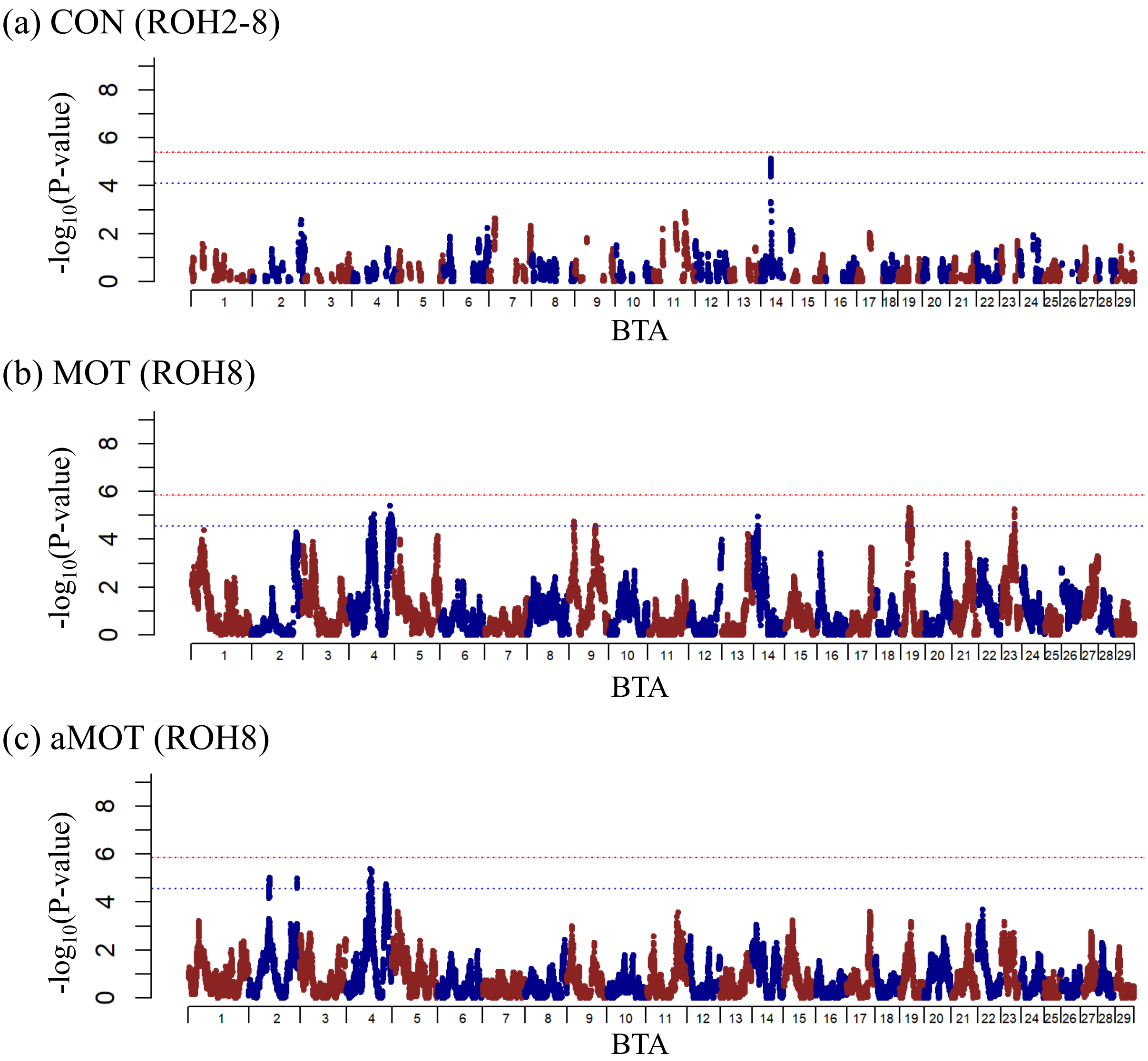

Figure S1. Genome-wide plots representing genome-wide suggestive association with (a) sperm concentration (CON), (b) sperm motility (MOT), and (c) MOT after freeze-thawing (aMOT) in Japanese Black bulls. Results of runs of homozygosity (ROH) of lengths 2–8 Mb (ROH2-8) and > 8 Mb (ROH8); x-axis indicates *Bos taurus* autosome (BTA) number, and y-axis indicates P-values ($-\log_{10}$). Horizontal red and blue lines represent genome-wide significant and suggestive thresholds, respectively.
