# Supplementary material for: Impact of Genome‐Wide and Regional Inbreeding on Semen Production Traits in Beef and Dairy Bulls
Source: Anim Sci J. 2025 Dec 3;96(1):e70138. doi: 10.1111/asj.70138 (PMC12675868; doi:10.1111/asj.70138)
Supplement: Supplementary file 2 — Figure S2: Genome‐wide plots representing genome‐wide suggestive association with (a) sperm motility (MOT) in Holstein bulls. Results of runs of homozygosity (ROH) of lengths of 2–8 Mb (ROH2–8); the x‐axis indicates Bos taurus autosome (BTA) number, and the y‐axis indicates p values (−log10). Horizontal red and blue lines represent the genome‐wide significant and suggestive thresholds, respectively. [file ASJ-96-e70138-s001.pdf]

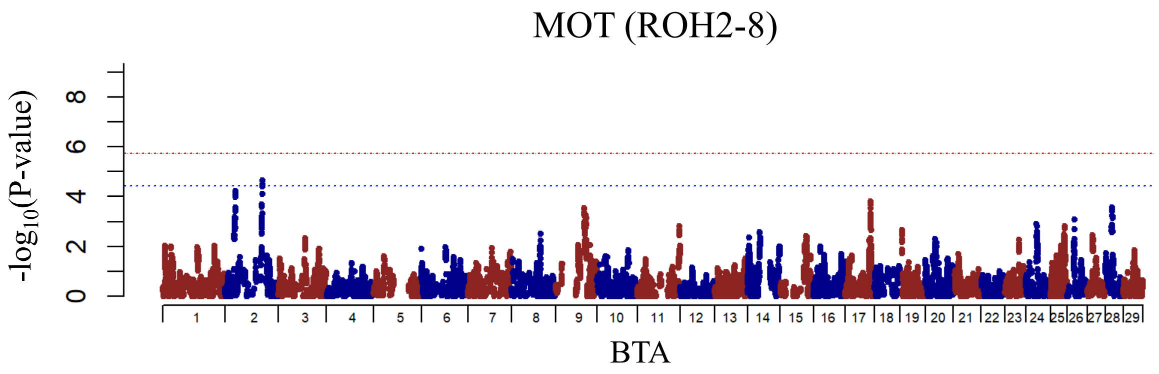

Figure S2. Genome-wide plots representing genome-wide suggestive association with sperm motility (MOT) in Holstein bulls. Results of runs of homozygosity (ROH) of lengths 2–8 Mb (ROH2-8); x-axis indicates *Bos taurus* autosome (BTA) number, and y-axis indicates P-values ($-\log_{10}$). Horizontal red and blue lines represent the genome-wide significant and suggestive thresholds, respectively.
