# Supplementary material for: Impact of Genome‐Wide and Regional Inbreeding on Semen Production Traits in Beef and Dairy Bulls
Source: Anim Sci J. 2025 Dec 3;96(1):e70138. doi: 10.1111/asj.70138 (PMC12675868; doi:10.1111/asj.70138)
Supplement: Supplementary file 3 — Table S1: Descriptive statistics of semen production traits in Japanese Black and Holstein bulls. [file ASJ-96-e70138-s003.pdf]

Table S1. Descriptive statistics of semen production traits in Japanese Black and Holstein bulls[a]

		Japanese Black				Holstein			
Traits	Abbreviations	Nid	Nrec	Mean	SD	Nid	Nrec	Mean	SD
Semen volume, mL	VOL	615	65,463	8.2	3.6	873	50,734	9.1	4.6
Sperm number, $\times 10^8$	NUM	615	65,463	87.2	39.1	873	50,734	101.3	59.9
Sperm concentration, $\times 10^8$/mL	CON	615	65,361	11.1	3.9	873	50,716	11.2	4.3
Sperm motility, %	MOT	615	64,728	79.0	5.2	868	49,909	79.1	6.5
Sperm motility after freeze-thawing, %	aMOT	615	59,344	41.7	6.9	795	41,772	42.2	7.4

[a]The results of descriptive statistics were previously reported by Nagai et al. (2022a). Nid: Number of animals. Nrec: Number of records.
